# Long-term effect of simplified dietary education on the nutritional status of patients after a gastrectomy

**DOI:** 10.1371/journal.pone.0252168

**Published:** 2021-05-21

**Authors:** Kyeong-Won Ryu, Jae-Moon Bae, Eun-Mee Kim, Ji Yeong An, Min-Gew Choi, Jun Ho Lee, Tae Sung Sohn

**Affiliations:** 1 Department of Dietetics, Samsung Medical Center, Seoul, Republic of Korea; 2 Division of Upper GI, Department of Surgery, Samsung Medical Center, Sungkyunkwan University School of Medicine, Seoul, Republic of Korea; Ohio State University Wexner Medical Center Department of Surgery, UNITED STATES

## Abstract

Dietary education is regarded as an important and useful tool for influencing nutritional status. Since long, dietary education has been performed to improve the nutritional status of patients after a gastrectomy. This study aimed to investigate the effect of simplified dietary education on the nutritional status of patients after a gastrectomy. A total of 1,150 patients with gastric cancer underwent surgery between March 2014 and October 2015 at the Samsung Medical Center (SMC). Of these, we used the case-control matching method (1:1 match) by stratifying the factors of age and sex and included 100 patients in each group. The clinicopathologic data of the patients for two years after the gastrectomy were prospectively collected and retrospectively analyzed. The educated group (ED, N = 100) was provided with a simplified, ordinary dietary education at regular outpatient clinic visits that occurred at 1, 3, 6, and 12 months after gastrectomy and at 1-year intervals thereafter. The clinicopathologic characteristics and nutritional parameters of the educated group (ED) (N = 100) and the non-educated group (NED) (n = 100) were compared. There were no significant differences between the two groups in terms of clinical characteristics and serological parameters. Nutritional parameters, which included body weight loss, body mass index (BMI) change, and prognostic nutritional index (PNI), were also not significantly different between the two groups. Simplified dietary education at regular outpatient clinic visits was ineffective in reducing weight loss after a subtotal gastrectomy. Further research or other methods may be needed to reduce weight loss after a gastrectomy.

## Introduction

It is well known that gastric cancer is the fourth most common malignant disease worldwide and the second most common cause of death from cancer, and is now the second most common cancer and the fourth most common cause of cancer death in South Korea [1–3]. Gastric resection with lymphadenectomy has been the mainstay gastric cancer treatment, although chemotherapy has prolonged the survival of patients with advanced disease [4]. As early detection and curative surgery increase in patients with gastric cancer in South Korea, the five-year survival rates of patients with early gastric cancer have also improved to ≥ 90%, which means that more patients will live longer after undergoing gastric resection [5].

Malnutrition is considered as a natural nutritional consequence of a gastrectomy, and malabsorption and malnutrition are prevalent in survivors of esophageal and stomach cancer, which might be underappreciated [6]. Kim et al. [1] reported that the high risk of malnutrition after gastrectomy might be attributed to inadequate oral consumption, malabsorption, rapid intestinal transit time, and loss of the reservoir function of the stomach [7].

Malnutrition has also been known as an independent risk factor for postoperative mortality and morbidity in major gastrointestinal surgery, with an increased incidence of postoperative complications [1,6]. Malnutrition manifests through weight loss or malabsorption. Despite being progressively evident with postoperative time in 15.6% to 48.9% of the patients, it is rarely considered as a serious problem. However, both gut and pancreatic insufficiency represent modifiable targets in an interdisciplinary approach to recovery [6]. Accordingly, it has become essential for clinicians to choose an appropriate form of nutritional therapy for patients recovering from gastric cancer surgery and to promote their rehabilitation [6,8].

The longer survival of patients with early gastric cancer after curative gastrectomy will increase the risk of malnutrition. These patients may experience decreased oral intake, weight loss, uncomfortable changes in dietary habits, dumping syndrome, or diarrhea, which may result in a limitation in daily life, general weakness, decreased social activity, and hospitalization in severe cases. Therefore, nutritional support is very important not only for long-term survival but also for the quality of life of patients after a gastrectomy [7].

Several procedures or methods have been used to improve the nutritional status of patients after a gastrectomy, such as the modification of reconstructive methods, increasing the volume of the remnant stomach, supplemental parenteral nutrition, changes in dietary menus or dietary habits, and recommendations for dietary education [9]. Various reconstructive methods, including increasing the volume of the remnant stomach, have not shown substantial benefits in nutritional parameters over those caused by other procedures [9–12]. Oral nutritional supplementation (ONS) was not effective in improving nutritional status after a gastrectomy [13].

Dietary education is regarded as an important and useful tool for influencing nutritional status. It has mostly been performed during the hospitalization period after a gastrectomy and at regular outpatient clinic follow-up visits after discharge.

Since long, dietary education has been performed after a gastrectomy to prevent nutritional depletion; however, its effect on nutritional changes has not been evaluated. Many hospitals have nutritional support teams that assess the nutritional status of patients and provide dietary support counseling, especially in gastrectomized patients.

We believe that dietary education has the advantage of being easy to perform and may have long-term effects. It decreases or prevents dumping syndrome, which may improve the patient’s quality of life. Above all, it is cost-effective and requires no specific instruments or medications. It may also contribute to a better relationship between doctors and patients. It is assumed that continuous dietary or nutritional education is very useful for the nutritional support of patients after a gastrectomy. Therefore, it is very important to prove the effect of dietary education after a gastrectomy for the improvement of dietary education.

However, few studies have examined the effect of dietary education after gastrointestinal surgery, which is rare, especially after a gastrectomy [14]. Accordingly, the present study aimed to elucidate the effect of simplified dietary education on the nutritional status of gastric cancer patients after a gastrectomy.

## Materials and methods

### Study population

A total of 1,150 patients with gastric cancer underwent surgery between March 2014 and October 2015 at the Samsung Medical Center (SMC). We selected patients who 1) underwent subtotal gastrectomy, 2) were diagnosed with pathologically confirmed Stage I (T1N0, T1N1, and T2N0) according to the American Joint Cancer Classification (AJCC; 8^th^ edition) and did not receive postoperative adjuvant chemotherapy that might influence nutritional status, and 3) had two years of body weight records and laboratory findings available. A total of 334 patients were selected serially, and all patients underwent a gastrectomy conducted by six surgeons at our institute.

**Fig 1:**
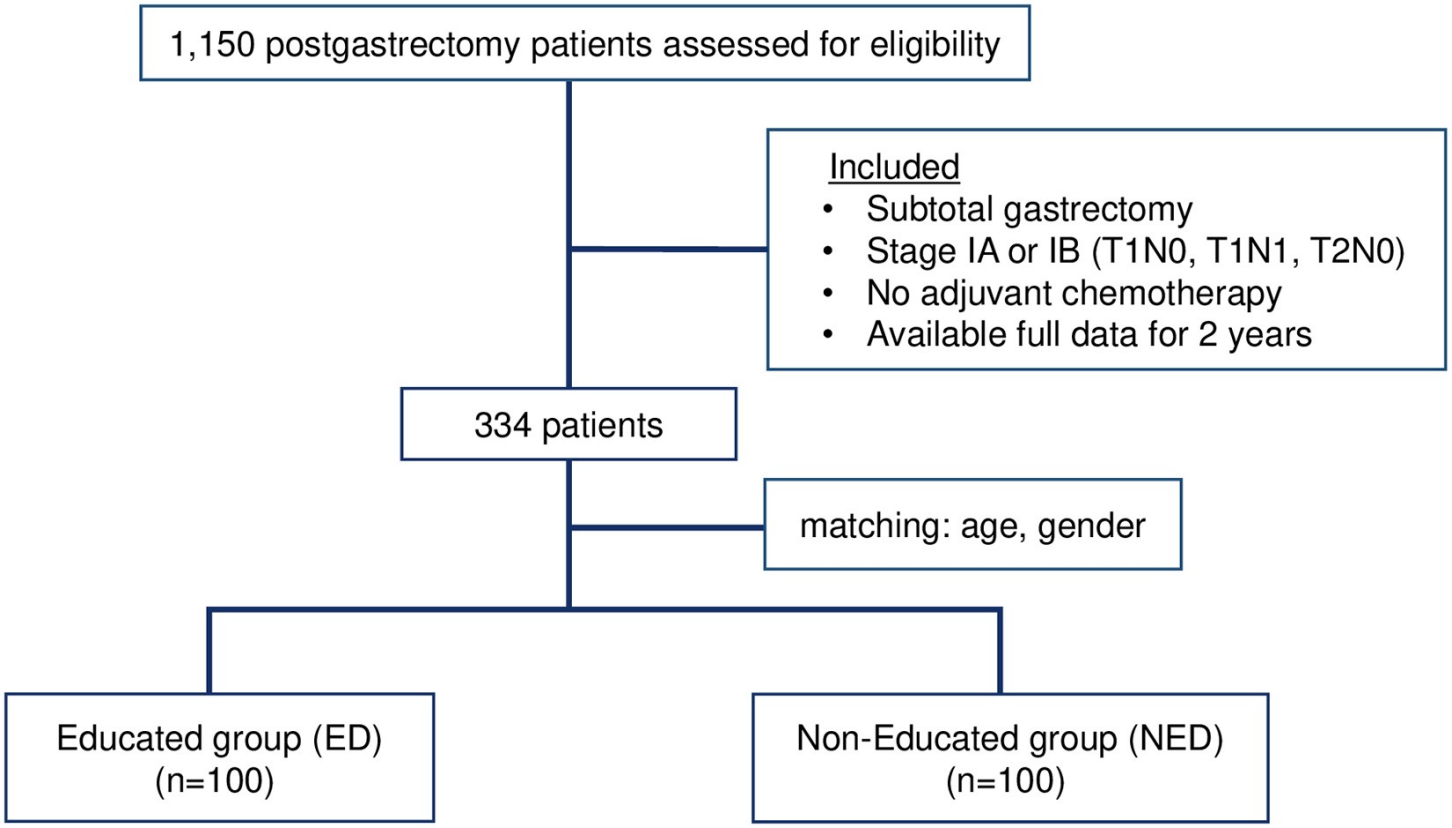
Scheme of the study.

Of the 334 patients, 101 patients from the educated group (ED) and 233 patients from the non-educated group (NED) were selected. We used the case-control matching method (1:1 match) to stratify the sample based on age and sex. Out of 101 patients, 100 from the ED group were serially included based on the selection criteria, and 100 patients from the 233 NED group were matched by age and sex. Finally, we included 100 patients in each group and statistically analyzed and compared the nutritional factors between the two groups (Fig 1).

Simplified dietary education is not a simple communication between doctors and patients. It is a set of learning experiences designed to facilitate the voluntary adoption of eating and other nutrition-related behaviors for health. Only one of the six surgeons checked body weight;, dietary habits including frequency, volume, and related symptoms;, and signs of dumping syndrome. Subsequently, the surgeon counseled the patients and their family about preventing nutritional deficits, and encouraged changes in dietary habits during regular outpatient clinic visits that occurred at 1, 3, 6, and 12 months after the gastrectomy and at 1-year intervals thereafter. The other five surgeons checked only body weight without paying attention to dietary education. Of the 334 patients, 101 and 233 were in the ED and NED groups, respectively.

The clinicopathological data of the patients for two years after a gastrectomy were prospectively collected in a database, which was then retrospectively reviewed and analyzed. Each patient was followed up for more than two years. The clinicopathological data included age, sex, type of surgery, anastomotic method, first day of flatus, length of hospital stay, complications, and disease stage. Nutritional parameters included weight change, body mass index (BMI), prognostic nutritional index (PNI), and total lymphocyte count (TLC).

### Ethical standards

All procedures followed were in accordance with the ethical standards of the responsible committee on human experimentation (institutional and national) and with the Helsinki Declaration of 1964 and all its amendments. This study did not include any minors. Ethical issues and patients’ consent were approved by Panel A (YK On, CH Park, YL Choi, HI Choi, DY Oh, BH Jeong, HJ Ahn, KA Kim, SY Chung, IG Kwon, HS Song, SH Yoo, CH Choi) of the Institutional Review Board of SMC (file no. 2019-07-139). Patients’ consent for retrospective analysis of medical records was routinely obtained before surgery as written consent or the equivalent (electronic document).

### Surgical procedure

All patients underwent a subtotal gastrectomy by a minimally invasive (laparoscopically/robot-assisted) procedure or open surgery, depending on the location of the tumor. Subtotal gastrectomy referred to distal subtotal gastrectomy, while reconstructive surgery included Billroth I, Billroth II, and Roux-en-Y anastomotic methods depending on the patient’s condition or the surgeon’s preference.

### Measurements

We analyzed the nutritional factors of the matched 200 patients and compared by comparing body weight, BMI, absolute lymphocyte count (ALC), serum albumin level, and PNI preoperatively and at 1, 3, 6, 12, and 24 months after the gastrectomy. We also analyzed the correlation between nutritional factors and compared the differences between the ED and NED groups. Postoperative surgical complications were graded according to the Clavien-Dindo (CD) classification [15,16].

### Statistical analysis

All statistical analyses were performed using SPSS (version 25.0; SPSS Inc., Chicago, IL, USA). Correlations between each group and the clinicopathological variables were analyzed using the Pearson’s correlation coefficient, chi-squared test, Student’s t-test, or Fisher’s exact test for categorical variables. All numerical data have been described as the standard error of the mean. A paired t-test was used to compare continuous variables, and p-values < 0.05 were considered statistically significant.

## Results

### Clinical characteristics

We used a 1 to 1 case-matching method to enroll all 200 patients retrospectively in the ED (N = 100) or NED (N = 100) groups. There were no significant differences between the two groups in age, sex, height, weight, BMI, cancer stage, and complication rates. There were significant differences in surgery type, anastomosis method, first day of diet, length of stay, and pathologic stage between the two groups. None of the patients had undergone adjuvant chemotherapy because they were all in the early stage. Most patients had an open surgery type, especially in the ED group, and the surgery type was equally distributed in the NED group. The NED group had more Billroth I type reconstructions (69%) than the ED group, while the ED group had more Billroth II reconstructions (82%) than the NED group. The first flatus was later in the ED group, and the length of hospital stay was longer in the ED group (Table 1).

**Table 1:** Clinicopathological characteristics of the patients.

Variables		Total (N = 200)	ED (N = 100)	NED (N = 100)	p-value
Age (years)		54.4 ± 10.1	54.6 ± 10.2	54.3 ± 10.1	0.851
Gender	M	122 (61%)	61 (61%)	61 (61%)	1.000
	F	78 (39%)	39 (39%)	39 (39%)	
Height (cm)		164.3 ± 8.0	164.2 ± 7.9	164.4 ± 8.3	0.871
Weight (kg)		66.0 ± 10.1	66.2 ± 10.0	65.9 ± 10.4	0.831
BMI (kg/m^2^)		24.4 ± 2.7	24.5 ± 2.7	24.3 ± 2.8	0.650
Surgery type (cases)	Laparoscopic	58 (29%)	4 (4%)	54 (54%)	< 0.001
	open	142 (71%)	96 (96%)	46 (46%)	
Anastomosis method	Billroth I	124 (62%)	38 (38%)	86 (86%)	< 0.001
	Billroth II	76 (38%)	62 (62%)	14 (14%)	
First day of diet (postoperative day)		4.83 ± 0.6	5.2 ± 0.5	4.5 ± 0.5	< 0.001
Length of hospital stay (postoperative day)		8.98 ± 5.6	10.28 ± 7.7	7.68 ± 1.2	0.001
Cancer stage^a^	Stage IA	188 (94%)	99 (99%)	89 (89%)	0.003
	Stage IB	12 (6%)	1 (1%)	11 (11%)	
Complication	Yes	10 (5%)	5 (5%)	5 (5%)	1.000
	No	190 (95%)	95 (95%)	95 (95%)	

Values are presented as mean ± standard error or number (%).

ED, educated group; NED, non-educated group.

^a^ 8th American Joint Committee on Cancer TNM staging system.

Student’s t-test and chi-square test.

Subject complications are summarized in Table 2. There were no significant differences in short-term postoperative morbidity between the two groups.

**Table 2:** Postoperative complications.

Group	Educated group (ED)	Non-educated group (NED)
Grade II	3	3
Wound problem	3	1
Delayed gastric Emptying or dumping syndrome	0	2
Grade II	2	1
Ileus	1	0
Mesenteric hernia	1	0
Fluid collection	0	1
Grade III	0	1
IIIa	0	0
IIIb	0	1
Grade IV	0	0
Total	5	5

Values are presented as numbers.

P > 0.05 between ED and NED group by Fisher’s exact test.

### Serological parameters (Alb, TLC)

No significant differences were observed in serum albumin levels and TLCs between the ED and NED groups at 3, 6, 12, and 24 months postoperatively. The average serum albumin concentration was 4.5 ± 0.3 g/dl in both group at admission and 4.5 ± 0.2 g/dl in the ED group and 4.5 ± 0.3 g/dl in the NED group at 24 months postoperative (p > 0.05). However, these parameters returned to admission levels regardless of the group at 24 months postoperative. The average TLC concentration was 2114.2 ± 689.8 x10^3^/μL in the ED group and 2217.0 ± 656.4 x10^3^/μL in NED group at admission, and 2031.6 ± 604.6 x10^3^/μL in the ED group and 2132.1 ± 674.1 × x10^3^/μL at 24 months postoperative (p > 0.05).

### Nutritional parameters (weight loss%, BMI, PNI)

**Fig 2:**
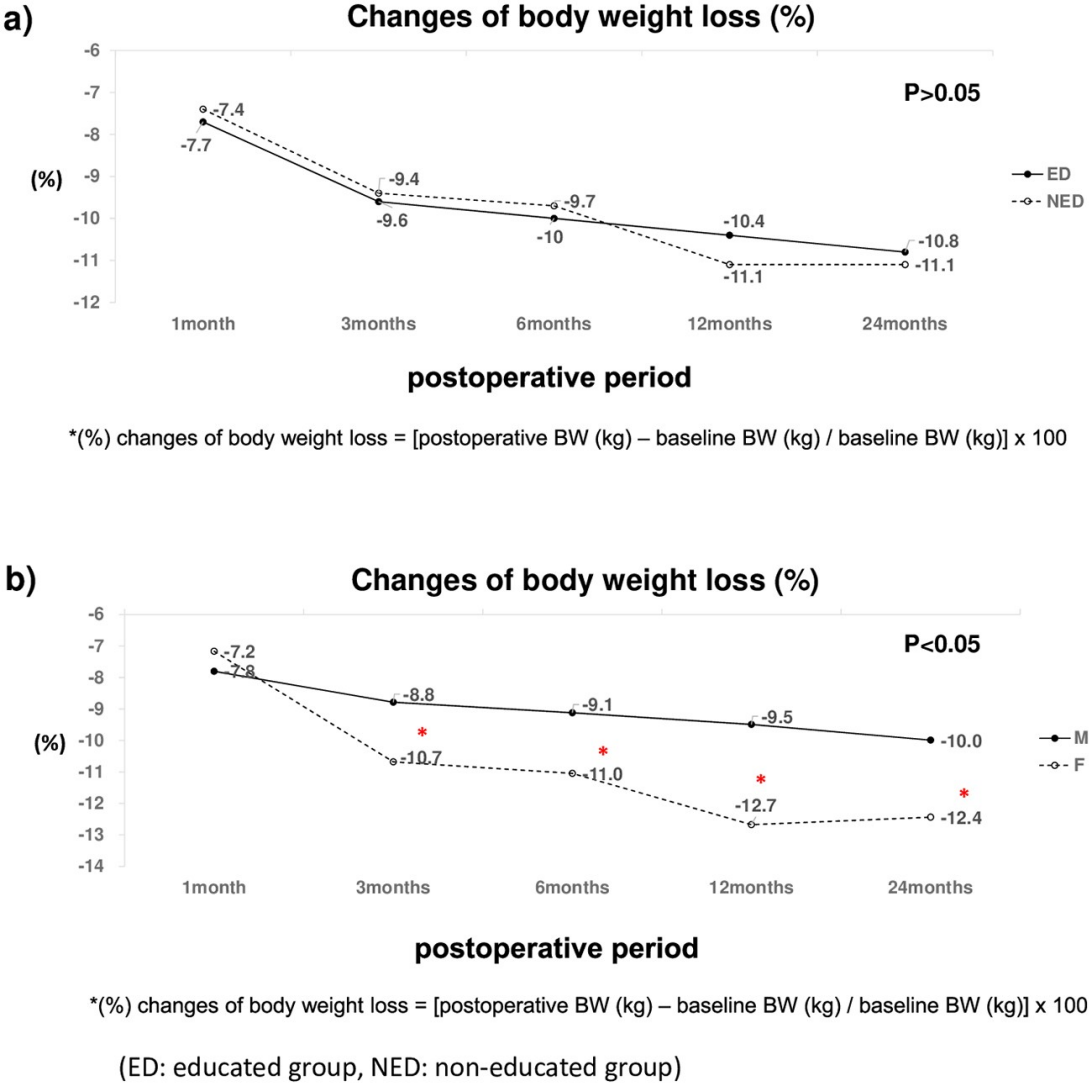
Changes in body weight loss (%) according to dietary education (a) and Gender (b). ED: Educated group; NED: Non-educated group.

**Fig 3:**
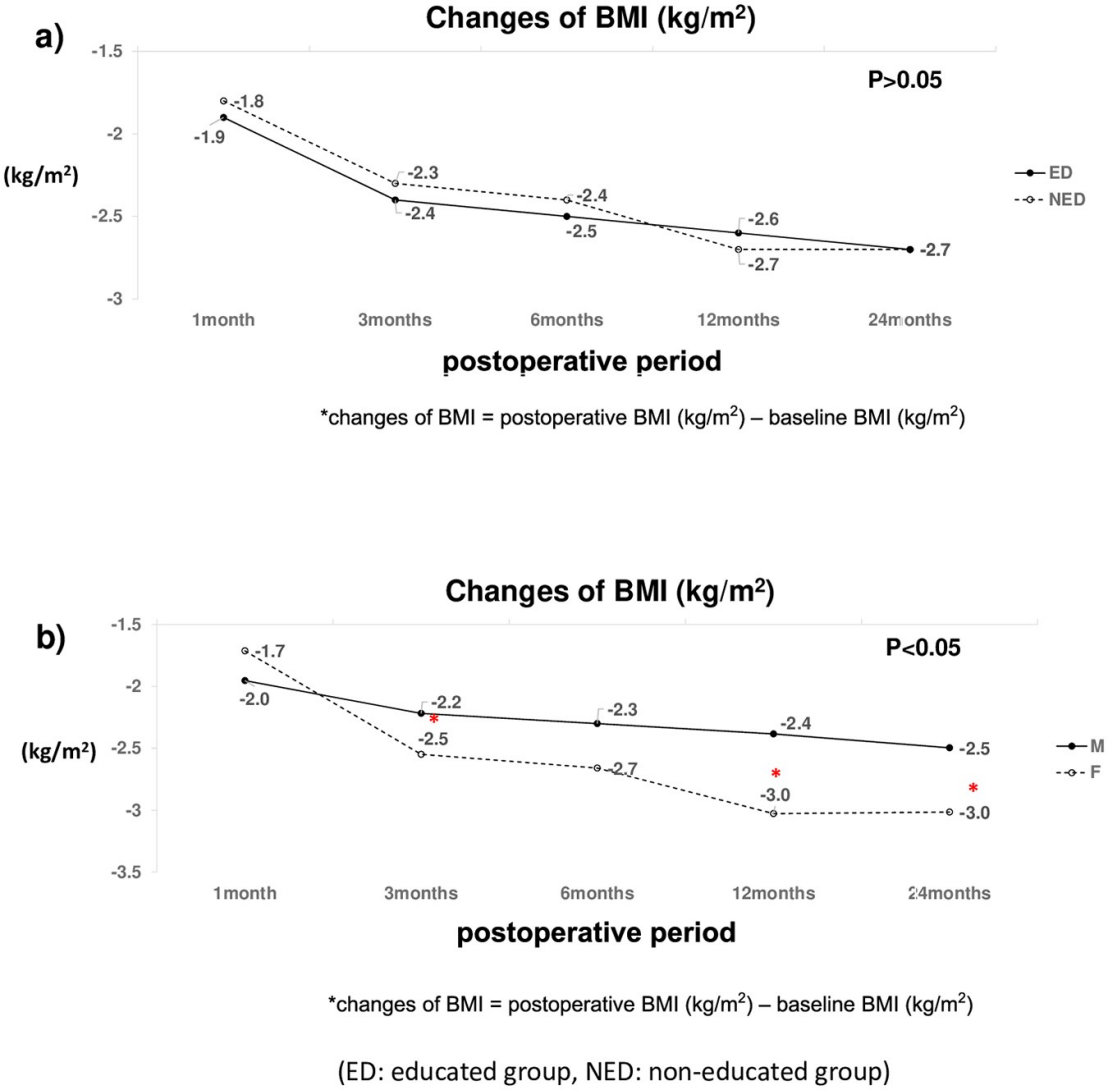
Changes in Body Mass Index (BMI) according to dietary education (a) and Gender (b). Changes in BMI, postoperative BMI (kg/m^2^), baseline BMI (kg/m^2^); ED: Educated group; NED: Non-educated group.

**Fig 4:**
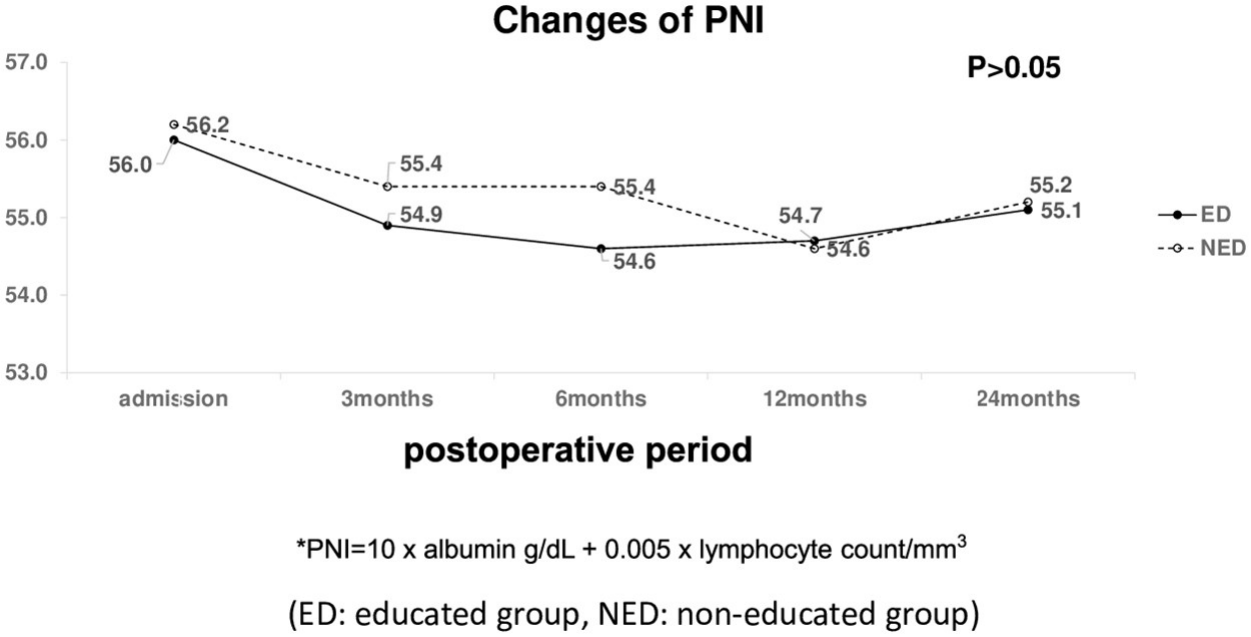
Changes of Prognostic Nutritional index* (PNI) according to dietary education. *PNI = 10 × albumin (g/dl) + 0.005 × lymphocyte count/mm^3^; ED: Educated group; NED: Non-educated group.

The nutritional parameters of the ED and NED groups over time were compared. Body weight loss, BMI change, and PNI did not differ significantly over time between the two groups. Trends in body weight loss, BMI change, and PNI between the groups have been shown in Figs 2–4. Although the nutritional parameters were not significantly different between the two groups, body weight loss and BMI significantly decreased over time in both (p< 0.001). Interestingly, females showed more weight loss than did males at 3,6,12, and 24 months postoperative, regardless of the group (p< 0.05) (Fig 2). The percent change in body weight was not significantly correlated with serum albumin or TLC.

## Discussion

It is well known that malnutrition after gastrointestinal surgery for cancer is closely related to poor prognosis and decreased quality of life. Previous studies showed that malnutrition was prevalent in gastric cancer patients preoperatively, which was related to postoperative morbidity, and that malnutrition before gastrectomy and at any time after surgery (at 1, 3, 6, or 12 months) significantly and adversely affected overall survival, suggesting that nutritional interventions to lessen the impact of postoperative malnutrition might offer hope for prolonged survival [17,18].

Post-gastrectomy syndrome, including dumping syndrome, with symptoms such as poor appetite, nausea, vomiting, diarrhea, and weight loss, may result in malnutrition. Some studies reported that post-gastrectomy syndrome resulted from the altered form and function of the stomach and that gastrectomy disrupted the reservoir capacity, mechanical digestion, and gastric emptying. The findings suggested that early recognition of symptoms with prompt evaluation and treatment was essential, and that many syndromes might be resolved with minimal intervention or dietary modifications. The study concluded that an integrated team approach to risk assessment, patient education, and postoperative management was critical to the optimal care of patients with gastric cancer [19].

It is very challenging to influence the dietary habits of patients to improve their nutritional status or to prevent malnutrition in surgical patients, especially after a gastrectomy.

Evidently, nutritional support is important for patients after a gastrectomy. Some methods, such as enteral supplementation, were introduced in the postoperative gastric surgery period but showed no significant benefit [13]. Some prospective intervention studies which used enteral immuno-nutrition also did not demonstrate the effectiveness of nutritional intervention during the perioperative period [20].

In our study, both the ED and NED groups showed similar patterns of change in nutritional parameters, such as weight loss, BMI, and PNI, at the time intervals analyzed after the gastrectomy, which suggested that simplified or ordinary dietary counseling or education was ineffective as nutritional support after a gastrectomy. The reason for the ineffectiveness of simplified dietary education was deduced to be the poor content of the dietary education, or problems in the delivery of the educational contents, such as a short education time, inadequate place, or insufficient educational tools.

The understanding or acceptance level of patients may also be problematic. Some patients might not trust the dietary recommendations or might not follow it. The present findings imply that changing one’s dietary habits might be difficult and it could be accompanied by family indifference. Furthermore, the quality of dietary counseling provided by the surgeon at the outpatient clinic visits may be poor or inadequate, which indicates the need for a specialized diet counseling team with an intensive or advanced program. The specialized diet counselor needs to be a dietician, so patients feel comfortable, and sufficient time and appropriate tools or materials should be provided. Counseling should be performed repetitively and feedback should be obtained regularly.

In our study, the ED group underwent more open-type surgeries and Billroth II type reconstruction according to the surgeon’s preference than the NED group. The NED group underwent more Billroth I reconstructions, which was the preferred type of minimally invasive surgery (laparoscopic or robotic-assisted gastrectomy). Although there were significant differences in surgery types and reconstruction methods between the two groups, there was no significant difference in nutritional parameters between the two groups. These findings indicate that the surgery type and reconstruction methods were not related to changes in nutritional parameters.

Complication rates were also not related to the surgery type or reconstruction method used in this study.

Interestingly, the present study found that female patients lost more weight and had poorer recovery after the gastrectomy than did male patients, regardless of dietary education. It was difficult to explain the reason for the difference. Possibly, female patients could not support themselves in their family or could not follow the dietary recommendations in their personal situations. The findings indicate that dietary education should focus more on female rather than male patients, and educational materials should be developed and customized for female patients.

Lee et al. [14] reported that intensive education was more effective than standard education is in improving nutritional status after a gastrectomy. However, this study has some limitations. First, the authors used a patient-generated subjective global assessment (PG-SGA) tool to assess participants’ diet, and measured self-efficacy and satisfaction with meals for three months following hospital discharge, which were more subjective and dependent upon the patient’s condition at the time of the interview. Second, they included a relatively small number of patients and followed them for only three months after the gastrectomy, which was too short to show a concrete effect of dietary education.

Our study may be the first to evaluate the effect of dietary education for patients after gastrectomy on a large scale with a long-term follow-up period. It was evident that a simplified dietary education at regular outpatient clinic visits was ineffective in reducing weight loss after a subtotal gastrectomy. Our study has some limitations. First, it was a retrospective study, although data were collected prospectively, and it was not a randomized study. Only one surgeon performed dietary education for his patients, which might have caused a selection bias. Second, this study included a relatively large number of gastric cancer patients but it was difficult to calculate a sample size to prove the effect because there was little evidence about dietary education after the gastrectomy. Finally, the educational methods or contents may be inadequate. It was also very difficult to find any methods or tools with sufficient reliability and validity in assessing whether the educated group of patients actually adhered to the dietary advice provided to them or they ignored and resorted to their own way of nutritional support. Our study suggests that further research should be conducted and a new educational modality or other intervention methods should be developed to improve the nutritional status of patients after a gastrectomy.
